# Rapid acquisition of daptomycin resistance in a *Corynebacterium striatum* osteoarticular infection: case report and discussion on antimicrobial resistance

**DOI:** 10.1128/asmcr.00106-25

**Published:** 2025-09-11

**Authors:** Ines Rezzoug, Emmanuel Zamparini, Charles Court, Assaf Mizrahi, Laurent Dortet, Cécile Emeraud

**Affiliations:** 1 Department of Bacteriology-Hygiene, Bicêtre Hospital, Assistance Publique-Hôpitaux de Paris 26930, Le Kremlin-Bicêtre, France; 2 Team 'Resist' UMR1184 'Immunology of Viral, Auto-immune, Hematological and Bacterial Diseases (IMVA-HB)', Faculty of Medicine, INSERM, Paris-Saclay University 89691 , Le Kremlin-Bicêtre, France; 3 Associated French National Reference Center for Antibiotic Resistance: Carbapenemase-Producing Enterobacterales, Le Kremlin-Bicêtre, France; 4 Department of Infectious Diseases, Bicêtre University Hospital, APHP 55662, Le Kremlin-Bicêtre, France; 5 Department of Orthopedic Surgery, Bicêtre Hospital, Assistance Publique–Hôpitaux de Paris 26930, Le Kremlin-Bicêtre, France; 6 Department of Clinical Microbiology, Saint-Joseph & Marie-Lannelongue Hospitals, Paris, France; 7 Micalis Institute UMR 1319, Université Paris-Saclay, INRAe, AgroParisTech 27048, Châtenay-Malabry, France; Rush University Medical Center, Chicago, Illinois, USA

**Keywords:** *Corynebacterium striatum*, daptomycin resistance

## Abstract

**Background:**

*Corynebacterium striatum*, a commensal of the skin microbiota, can cause invasive osteoarticular infections that are challenging to diagnose and treat due to biofilm formation. Daptomycin, owing to its potent activity against gram-positive bacteria and favorable toxicity profile, is frequently used as empirical therapy in the management of postoperative bone and joint infections. However, cases of acquired resistance remain rare and poorly understood.

**Case Summary:**

We describe a case of chronic T11–T12 spondylodiscitis in a paraplegic patient. During the first surgical intervention, a daptomycin-susceptible *C. striatum* isolate (CORY-1) was recovered. The patient received 14 days of daptomycin monotherapy (10 mg/kg). At the time of the second surgery, a daptomycin-resistant *C. striatum* strain (CORY-2) was isolated. Whole-genome sequencing revealed only 13 single nucleotide polymorphisms between the two isolates, including a nonsense mutation in *pgsA*, likely responsible for resistance. Additional mutations in *mltG* and *ftsI* genes may contribute to the modest increases observed in β-lactam minimum inhibitory concentrations.

**Conclusion:**

This case highlights the risk of rapid daptomycin resistance emergence in *C. striatum* under selective pressure. Daptomycin should be used with caution for long-term infections caused by this species. Alternative agents such as vancomycin or oxazolidinones may offer more reliable therapeutic options.

## INTRODUCTION

*Corynebacterium striatum* is a gram-positive bacillus and a commensal organism of the skin flora, but it can also cause invasive infections (1). This species has been implicated in various osteoarticular infections, including osteomyelitis, septic arthritis, and prosthetic joint infections (2–4). True *C. striatum* infections are difficult to diagnose, as several positive samples are necessary to rule out the possibility of contamination. Furthermore, treatment is often difficult due to the ability of *C. striatum* to form biofilms that protect the bacteria and contribute to its persistence (5). Vancomycin is often the antibiotic of choice to treat *C. striatum* infections. However, other antibiotics, such as linezolid, tetracyclines, or daptomycin, have shown low minimum inhibitory concentrations (MICs), suggesting potential effectiveness against this pathogen (2).

Daptomycin is a cyclic lipopeptide antibiotic that is bactericidal and active against gram-positive bacilli. It possesses efficacy comparable to that of vancomycin but with a lower toxicity profile, making it a preferred treatment option (1). Its bactericidal activity relies on a unique mechanism of action involving membrane disruption. In the presence of calcium ions (Ca^2+^), daptomycin binds to the bacterial plasma membrane, composed mainly of phospholipids, leading to the formation of an ion channel. This causes membrane depolarization and potassium efflux, ultimately resulting in cell death.

Daptomycin resistance has been described in *Staphylococcus*, *Enterococcus,* and *Corynebacterium* species (6). In *Staphylococcus*, resistance to daptomycin is due to the accumulation of negatively charged phospholipids on the cell membrane surface, leading to electrostatic repulsion of the daptomycin molecule. This phenomenon results from an accumulation of mutations in several genes, including *mprF*, *cls2*, and *pgsA* (7, 8). In contrast, in *Enterococcus* species, daptomycin resistance is primarily driven by mutations in *cls* and *gdpD* genes, which alter membrane composition and may lead to cell wall thickening, or by mutations affecting the LiaFSR regulatory system, which governs the cell envelope stress response (9, 10). As with *Staphylococcus*, the development of daptomycin resistance in *Enterococcus* typically requires an accumulation of mutations rather than a single genetic change.

Here, we report the rapid acquisition of high-level daptomycin resistance in a *C. striatum* strain isolated from a patient with an osteoarticular infection, who was initially treated with daptomycin following orthopedic surgery.

## CASE PRESENTATION

**Fig 1:**
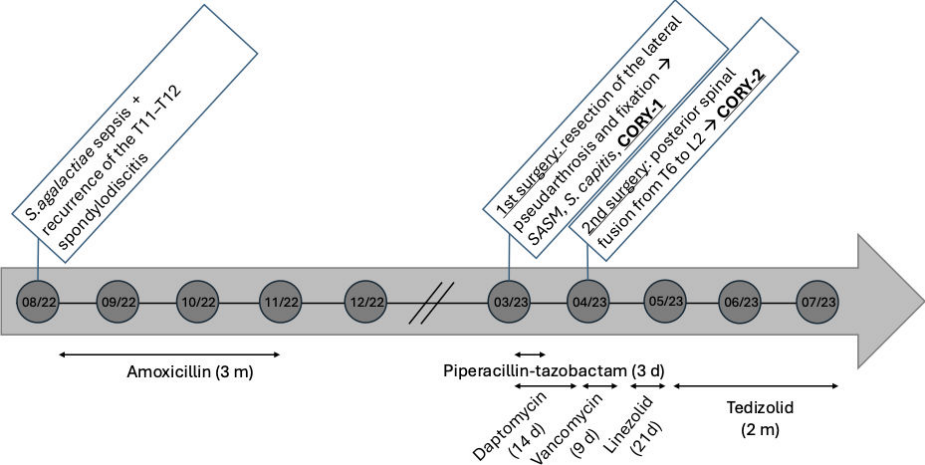
Chronological representation of antibiotic therapy and orthopedic surgical interventions from August 2022 to July 2023.

A 49-year-old man was hospitalized in August 2022 for surgical management of chronic T11–T12 spondylodiscitis, associated with multiple rib lesions and bilateral pleural involvement. His medical history included paraplegia following a road traffic accident in 1993, with a subsequent development of a Charcot spine at T12–L1, complicated by septic pseudarthrosis secondary to multiple pressure ulcers. The initial spinal instrumentation placed after the 1993 accident was removed in 2002 due to infection. In 2014, the patient was treated for costal osteitis related to a subscapular pressure ulcer, with *S. aureus* and *Proteus mirabilis* identified from intraoperative samples. Management included surgical debridement and a 6 week course of antibiotic therapy with rifampin and levofloxacin. In 2019, the patient developed T11–T12 spondylodiscitis in the context of a new ischial pressure ulcer. *S. epidermidis* was isolated and treated with rifampin and doxycycline for 3 months. In the summer of 2022, the patient presented with fever. Blood cultures were positive for *S. agalactiae*, and imaging confirmed a recurrence of the T11–T12 spondylodiscitis, originating from the ischial pressure ulcer and complicated by associated rib and pleural involvement, treated by a 3 months course of antimicrobial therapy with amoxicillin (Fig. 1). Due to the need for both anterior and posterior surgical approaches as well as thoracic intervention, the patient was referred for the first time to our teaching hospital. In the spring of 2023, the first stage involved resection of the lateral pseudarthrosis and fixation. One month later, the second stage consisted of a posterior spinal fusion from T6 to L2, using bone graft harvested from the right iliac crest. Between the two surgical stages, the patient received intravenous antibiotic therapy consisting of piperacillin–tazobactam (4g × 3 /day IV) and high-dose daptomycin (10 mg/kg in a single daily infusion).

All first perioperative samples grew methicillin-susceptible *S. aureus*, methicillin-resistant *S. capitis,* and *C. striatum* susceptible to daptomycin (named CORY-1). Based on these results, piperacillin-tazobactam was shortly discontinued, and daptomycin was maintained in monotherapy until the second-stage surgery. After the second procedure, all five perioperative bone samples were positive for *C. striatum* resistant to daptomycin (named CORY-2). The antimicrobial therapy was immediately modified following the second intervention, and vancomycin was initiated. In light of the microbiological results and the favorable clinical and biological evolution, treatment was switched to oral linezolid after 10 days of intravenous vancomycin. After 3 weeks of linezolid therapy, the treatment was discontinued due to anemia and replaced with tedizolid. Under tedizolid, the anemia regressed, and the treatment was carried out to completion, for a total duration of 3 months.

### Antimicrobial susceptibility testing 

MICs for amoxicillin, meropenem, and imipenem were determined using E-test strips (BioMérieux) (Table 1), while MICs for vancomycin, teicoplanin, daptomycin, and dalbavancin were assessed by broth microdilution using Sensititre plates (Thermofisher, Dardilly, France), according to manufacturer’s recommendations. All MIC determinations were performed using a 0.5 McFarland standard inoculum. These tests were performed for both clinical *C. striatum* isolates (CORY-1 and CORY-2). Comparing the two isolates, we observed notable increases in MIC values: meropenem rose from 0.19 mg/L in CORY-1 to 0.75 mg/L in CORY-2, imipenem from 0.75 mg/L to 1.5 mg/L and vancomycin from 0.25 mg/L to 0.5 mg/L. Most strikingly, the MIC for daptomycin increased dramatically, from 0.12 mg/L to >32 mg/L (Table 1).

**TABLE 1:** MICs for β-lactams and glycopeptides for the two strains of *C. striatum* CORY-1 and CORY-2 isolated December 2022 and April 2023, respectively

Antibiotic	MIC (mg/L)		EUCAST interpretation
	March 2023 CORY-1	April 2023 CORY-2	
Amoxicillin	6	6	R
Meropenem	0.25	0.75	No data
Imipenem	0.75	1.5	No data
Piperacillin-tazobactam	>256	>256	No data
Vancomycin	0.25	0.5	S
Teicoplanin	0.12	0.12	No data
Daptomycin	0.12	>32	No data
Dalbavancin	0.015	0.015	No data
Linezolid	0.25	0.25	S
Tedizolid	0.12	0.12	S

### Whole genome sequencing

To understand the mechanism involved in daptomycin resistance in these *C. striatum* isolates, the two strains CORY-1 and CORY-2 were compared using genomic analysis through whole genome sequencing (WGS). The genomes of the two isolates were sequenced using Illumina short-read sequencing (NextSeq500 system, Illumina technology). Reads were assembled using Shovill v1.1.0 and SPAdes v3.14.0. To assess whether this recurrence came from the same bacterial strain or a completely independent infection, we analyzed the number of single nucleotide polymorphisms (SNPs) between the two strains using SNIppy v6.4.0. and found only 13 SNPs between the two strains. Among these 13 mutations, only three resulted in amino acid changes, each affecting three different genes (*mltG*, *ftsl,* and *pgsA*), while the remaining mutations were silent. The *pgsA* gene, which encodes phosphatidylglycerophosphate synthase involved in the synthesis of phospholipid acids in the bacterial membrane, harbored a G147D mutation. The *mltG* gene, which encodes an endolytic murein transglycosylase involved in peptidoglycan synthesis by acting as an elongation terminator, carried the T102A substitution. The *ftsl* gene, encoding penicillin-binding protein PBP3, a key player in cell division and peptidoglycan synthesis, carried an A370D substitution.

## DISCUSSION

Acquired resistance to daptomycin in *Corynebacterium* species has been increasingly reported in recent years, particularly in *C. striatum*. Several clinical case reports and *in vitro* studies have described the ability of this organism to develop high-level resistance during antimicrobial therapy (11–13). Both clinical and experimental data have shown that resistance can emerge in 30–100% of isolates, depending on the strain and duration of treatment (6, 13), highlighting the capacity of this species to adapt under antibiotic pressure.

Our case provides additional evidence of this phenomenon. A patient treated for a chronic low-grade osteoarticular infection received 4 weeks of daptomycin therapy before a second isolate of *C. striatum* was recovered. Two clonally related isolates obtained 4 months apart exhibited an important increase in daptomycin MICs, from 0.12 mg/L to >32 mg/L. WGS confirmed that the two isolates, CORY-1 and CORY-2, were highly related, differing by only 13 SNPs, supporting the hypothesis of *in vivo* selection of resistance rather than reinfection by a distinct strain.

Three nonsynonymous mutations were identified between these two isolates. Among them, a nonsense mutation in *pgsA*, a gene encoding phosphatidylglycerol synthase, has previously been shown to confer high-level daptomycin resistance in *Staphylococcus*, *Enterococcus*, and *Corynebacterium* species (6, 14). Unlike *Staphylococcus* or *Enterococcus*, which typically require multiple mutations to achieve resistance (14), a single mutation in *pgsA* is sufficient in *C*. *striatum*. The G147D mutation identified in our case introduces a premature stop codon in the active site of the PgsA enzyme, abolishing phosphatidylglycerophosphate synthase activity. This results in a loss of phosphatidylglycerol, the primary target of daptomycin, thereby conferring resistance that has already been characterized (13). This mutation is consistent with previous reports: a recent systematic review investigating high-level daptomycin resistance in *C. striatum* showed that resistance typically emerged after 8–41 days of daptomycin therapy, and when explored, mutations in the *pgsA2* gene were systematically involved (15). To date, no specific mutations have been consistently associated with daptomycin resistance in other *Corynebacterium* species.. However, mutations in the *pgsA2* gene, previously linked to high-level resistance in *C. striatum*, have recently been described in other *Corynebacterium* spp., suggesting a potentially shared mechanism (16).

Two additional mutations were detected in *mltG* and *ftsI*, genes involved in peptidoglycan metabolism and cell division, respectively. These may contribute to the modest increase in β-lactam MICs observed in CORY-2, although their functional impact remains to be experimentally validated. Their presence may reflect compensatory mechanisms or adaptive cell envelope remodeling under selective pressure.

Together, these findings illustrate how a commonly used empiric regimen, although initially appropriate, may favor the selection of daptomycin-resistant subpopulations, especially in the context of chronic infections requiring prolonged empirical coverage and delayed microbiological diagnosis. This case underscores the need to reassess the use of daptomycin monotherapy in suspected *C. striatum* infections and to consider alternative agents or combination therapies early in the course of treatment.

To conclude, this case highlights the fact that, contrary to *Staphylococcus* spp. related infections, daptomycin should not be the antibiotic for *C. striatum* infections, especially in the case of infections requiring long-term treatment. Alternative agents, such as vancomycin or oxazolidinones, may represent a more reliable therapeutic alternative.

## Data Availability

Sequence data generated for this study are publicly available in GenBank under BioProject PRJNA1268192.
